# Association between wet-bulb globe temperature and epilepsy: a space-time-stratified case-crossover study in Taiwan

**DOI:** 10.1186/s41182-025-00755-z

**Published:** 2025-05-20

**Authors:** Yu-Tzu Chang, Yu-Ting Lin, Bao-Ru Chuang, Wen-Hsuan Chuang, Bing-Fang Hwang, Chau-Ren Jung

**Affiliations:** 1 Division of Pediatric Neurology, China Medical University Children’s Hospital, Taichung, Taiwan; 2 School of Post Baccalaureate Chinese Medicine, China Medical University, Taichung, Taiwan; 3 Department of Medical Research, China Medical University Hospital, Taichung, Taiwan; 4 Big Data Center, China Medical University Hospital, China Medical University, Taichung, Taiwan; 5 Department of Occupational Safety and Health, College of Public Health, China Medical University, No 91 Hsueh-Shih Rd, Taichung, 40402 Taiwan R.O.C.; 6 Department of Public Health, College of Public Health, China Medical University, No 91 Hsueh-Shih Rd, Taichung, 40402 Taiwan R.O.C.; 7 Department of Occupational Therapy, College of Medical and Health Science, Asia University, Taichung, Taiwan; 8 Japan Environment and Children’s Study Programme Office, National Institute for Environmental Studies, Tsukuba, Japan

**Keywords:** Climate change, Epilepsy, Space–time-stratified case-crossover study, Wet-bulb globe temperature

## Abstract

**Background:**

Epilepsy is a common neurological disorder characterized by an enduring predisposition to generate epileptic seizures, along with its neurobiological, cognitive, psychological, and social consequences. Although a few studies have assessed the associations of meteorological factors, such as temperature, atmospheric pressure, and relative humidity, with epilepsy, their findings remain inconsistent. In this study, we used the wet-bulb globe temperature (WBGT), an integrated heat stress index closely aligned with human thermal perception, to assess its associations with epilepsy risk.

**Methods:**

This study employed a space–time-stratified case-crossover study design, analyzing 187,657 epileptic seizures recorded in emergency department visits in the Taiwan Health Insurance Research Database between 2007 and 2017. Daily WBGT values at individuals’ residential addresses were estimated using a 1-km resolution spatiotemporal model. The effects of an interquartile range (IQR) increase in WBGT on the day of epileptic seizures were compared to 3–4 reference days within the same month using conditional logistic regressions combined with distributed lag non-linear models (DLNMs).

**Results:**

The lag-response relationship indicated a significant positive association between WBGT and epilepsy risk at lag 0 day (odds ratio [OR] = 1.083, 95% confidence interval [CI]: 1.061–1.105), whereas significant negative associations were observed at lag 1 and lag 2 day, suggesting a harvesting effect. The cumulative effect of heat persisted for 2 days. Additionally, the exposure–response relationship between WBGT and epilepsy at lag 0 day was linear, with no apparent threshold observed.

**Conclusion:**

Our findings suggest that heat exposure may trigger epilepsy, resulting in short-term clustering of epilepsy cases. As precision medicine continues to gain prominence, incorporating precise heat stress indicator, such as WBGT, into individualized epilepsy management strategies may enhance patient care and seizure prevention.

**Supplementary Information:**

The online version contains supplementary material available at 10.1186/s41182-025-00755-z.

## Background

Climate change is one of the most significant challenges impacting global health. Rising global average temperatures due to climate change contribute to an increasing burden of disease [1]. Global warming leads to several adverse effects, including more frequent and severe extreme heat events, the expansion of vector-borne infectious diseases, and heightened social and economic stress and disruption. These factors will have substantial negative consequences for various aspects of health care, including epilepsy [2, 3]. Further investigation and targeted research are needed to accurately quantify and predict health impacts of global warming.

Epilepsy is a common neurological disorder characterized by an enduring predisposition to generate epileptic seizures, along with its various neurobiological, cognitive, psychological, and social consequences [4]. Its diagnosis requires the occurrence of at least one epileptic seizure [4]. The underlying mechanisms of epilepsy may be complex, involving both intrinsic and extrinsic factors. Intrinsic factors, such as the underlying cause of epilepsy and individual physiology, along with extrinsic factors, including ambient temperature, humidity, and sunlight exposure, can significantly influence seizure occurrence [5, 6].

Clinical and animal studies suggest a threshold effect of body temperature on febrile seizures at the onset of convulsions [7]. In conditions like Dravet syndrome (DS), elevated body temperature, whether induced by fever, warm baths, ambient temperature, or physical exercise, is a well-recognized seizure precipitant [8]. Extrinsic factors, including environmental factors or endogenous factors, may transiently lower the seizure threshold. Meanwhile, triggering factors involve chemical or physiological stimulation capable of precipitating an ictal event [9].

Previous studies have investigated the associations between meteorological factors, such as air pressure, precipitation, relative humidity, sunshine duration, temperature, and wind velocity, and seizures occurrence (summarized in Table S1) [6, 10–15]. Among these factors, ambient temperature has received the most attention [14]. Studies conducted in Switzerland, Germany, Portugal, and Taiwan have reported significant negative association, suggesting a protective effect between ambient temperature and seizure occurrence [6, 10–12, 15]. Conversely, studies from Brazil and China have indicated positive associations, implicating increased temperatures as a seizure risk factor [13, 14]. However, temperature alone does not fully capture the complexity of heat stress experienced by individuals. Other meteorological factors, including relative humidity, solar radiation, and wind velocity, also play important roles in modulating heat stress levels [16]. The discrepancies in findings across previous studies may be attributed to regional variability in these meteorological factors, as well as differences in study design and analytical approaches. While some studies have explored the associations between relative humidity and seizures, their findings remain inconsistent (Table S1). Notably, none of the existing studies have simultaneously accounted for temperature, relative humidity, solar radiation, and air velocity to better quantify heat stress. This highlights the need for an integrated assessment of heat stress to understand its effects on seizure occurrence.

To address this critical knowledge gap, we aimed to investigate whether heat stress could trigger seizures. This study could help elucidate the mechanisms of epilepsy and inform the development of preventive measures to mitigate seizure episodes. The wet-bulb globe temperature (WBGT), an integrated heat index that accounts temperature, humidity, solar radiation, and air velocity, is a more comprehensive indicator of heat stress experienced by humans compared to ambient temperature. We conducted a space–time-stratified case-crossover study to examine the possible threshold effect of WBGT on epilepsy occurrence, utilizing high temporal resolution WBGT estimates generated through a meteorology-based hybrid model.

## Methods

### Study population

The data were sourced from the National Health Insurance Research Database (NHIRD), managed by the Health and Welfare Data Center (HWDC), Taiwan’s Ministry of Health and Welfare (MOHW). The HIRD contains individual-level information on beneficiary registration, ambulatory care claims, and inpatient claims, covering 99.9% of the population in Taiwan [17]. To protect individual privacy, the names of individuals, health care providers, and medical institutions in the NHIRD have been encrypted with unique and anonymous identifiers [17].

Prior to 2016, the NHIRD utilized the International Classification of Diseases, Ninth Edition, Clinical Modification (ICD-9-CM) for disease diagnosis, transitioning to the Tenth Edition (ICD-10) in 2016. The accuracy of epilepsy diagnoses in the NHIRD has been validated in a previous study, which reported the sensitivity and specificity of identifying epilepsy cases as 81.39% and 99.83%, respectively [18].

### Outcome of interest

Epilepsy was defined as an individual receiving at least two consecutive diagnoses coded by a physician using ICD-9-CM code 345 before 2016 and ICD-10 codes G40 and G41 since 2016. Epileptic seizures were identified form emergency department (ED) visits in the NHIRD between 2007 and 2017.

### Study design

A space–time-stratified case-crossover study was conducted to assess the association between WBGT and epilepsy occurrence [19]. In this design, each participant serves as their own control, with the WBGT conditions before or after a seizures attack event (case period) compared to a reference period. This study design effectively controls for potential confounding factors, including sex, age, socioeconomic status, smoking history, occupational history, and genetic predisposition [20]. The space–time-stratified approach restricts control days to the same day of the week within the same month and year, and at the same location (residential address) as the case day. For instance, if an event occurred on the first Tuesday of February 2008 (February 5, 2008), all other Tuesdays in February 2008 (February 12, 19, and 26, 2008) were used as the reference period. This approach ensures that each case is matched with three or four controls, minimizing bias related to seasonal and weekly variation, as well as spatial variation [19, 21].

### Exposure assessment

Meteorological variables, including maximum ambient temperature, relative humidity, wind speed, and solar radiation, were used to calculate the daily WBGT value at a 1 km × 1 km spatial resolution in Taiwan during the study period [22]. The maximum ambient temperature was obtained from the Taiwan Climate Change Projection and Information Platform (TCCIP) (https://tccip.ncdr.nat.gov.tw/). Air temperature at 2 m, dew point temperature at 2 m, zonal/meridional wind at 10 m, and surface solar radiation downward were sourced from the Reanalysis Interim (ERA-Int) of the European Centre for Medium-Range Weather Forecasts (ECMWF). Relative humidity was derived using air temperature and dew point temperature [23], while wind speed was estimated from zonal and meridional wind at 10 m [24].

First, maximum ambient temperature was treated as dry-bulb temperature. Dry-bulb temperature, wind speed, and solar radiation were incorporated into the following formula to calculate globe temperature [25]:

$${\text{T}}_{g}={T}_{d}+ 0.017\times S-0.208\times U,$$

where T_g_ is the globe temperature; T_d_ is the dry-bulb temperature (°C) (here using maximum ambient temperature); S is solar radiation (W/m^2^), and U is wind speed (m/s). The empirical expression using the Stull (2011) method to assess the wet-bulb temperature is as follows:

$${T}_{w}={T}_{d}\,\text{atan}\left[0.151977{\left(RH\%+8.313659\right)}^\frac{1}{2}\right]+\text{atan}\left({T}_{d}+RH\%\right)-\text{atan}\left(RH\%-1.676331\right)+0.00391838{\left(RH\%\right)}^\frac{3}{2}\text{atan}\left(0.023101RH\%\right)-4.686035,$$

where T_w_ is the wet-bulb temperature (°C) and RH is relative humidity. Finally, wet-bulb temperature, globe temperature, and dry-bulb temperature were used to estimate daily WBGT, using the following formula:

$$\text{WBGT}=0.7{T}_{w}+0.2{T}_{g}+0.1{T}_{d}.$$

To investigate the lagged effects of WBGT on epilepsy occurrence, daily WBGT values were assigned to individuals based on their residential addresses, and WBGT values for individual lagged days 0 to 21 were calculated.

### Covariates

Age and socioeconomic status (SES) were obtained from the registry for beneficiaries in the NHIRD. Age was categorized into four groups: < 20, 20–39, 40–64, and ≥ 65 years. SES was determined based on the monthly insured payroll-related amount and classified into four categories: ≤ 25th percentile, 25th–50th percentile, 50th–75th percentile, and ≥ 75th percentile.

### Statistical analysis

A conditional logistic regression combined with a distributed lag non-linear model (DLNM) was utilized to evaluate both the lag–response and exposure–response relationships between WBGT and epilepsy [27]. The DLNM framework incorporates a cross-basis function, enabling the simultaneous modeling of lag effects and exposure-response relationships. The reference WBGT level was set at 21.0 °C, corresponding to the 25th percentile of WBGT. The selection of lag periods, spline types, and the degree of freedom (df) was evaluated using the Akaike information criterion (AIC). Lag periods ranging from 0–3 to 0–10, and 0–21 days were tested based on previous studies [6, 14, 15]. The lag-response function for each lag period was modeled using B-splines with 4–7 df and natural cubic splines with 3–7 df. Additionally, the exposure-response function was modeled using linear terms, B-splines with df of 4–7, or natural cubic splines with df of 3–7. Based on the lowest AIC value, the optimal lag period was identified as 0–7 days, with the lag-response and exposure-response relationships modeled using a B-spline with 6 df and a linear term, respectively (AIC = 553,379.4; Table S2).

Sensitivity analyses were further conducted to assess the robustness of the findings by varying the lag periods (0–7, 0–10, and 0–21 days) and adjusting the spline types, including using a B-spline with 4 df for the exposure–response effect and a B-spline with 5 df for the lag-response effect. Additionally, stratified analyses by sex, age group, and SES were performed to identify heat vulnerable subpopulations. Results for both individual lag effects and cumulative lag effects were reported as odds ratios (ORs) and 95% confidence intervals (CIs) per interquartile change (IQR) increase in WBGT.

All statistical analyses were conducted using SAS version 9.4 (proc univariate and proc logistic) and R version 4.5.0 (survival and dlnm packages).

## Results

### Characteristics of the study population

A total of 187,657 ED cases with epilepsy were identified in our study population from January 1, 2007, to December 31, 2017. Table 1 summarizes the demographic characteristics of ED visits for epilepsy during the study period. The majority of epilepsy cases were male (67.3%), from lower SES groups (43.8%), and aged 40–65 years (41.5%).

**Table 1:** Demographic characteristics of epilepsy events (*n* = 187,657) from 1 January 2007 to 31 December 2017 in Taiwan

	Variables	Number of events (%)
Gender	Male	126,407 (67.4%)
	Female	61,250 (32.6%)
Age	< 20 years old	15,832 (8.4%)
	20–39 years old	60,559 (32.3%)
	40–64 years old	77,898 (41.5%)
	≥ 65 years old	33,368 (17.8%)
Socioeconomic status	≤ $15,840 NTD	82,136 (43.8%)
	$15,840–$19,200 NTD	50,037 (26.7%)
	$19,201–$27,600 NTD	35,817 (19.1%)
	> $27,600 NTD	19,667 (10.4%)

**Fig. 1:**
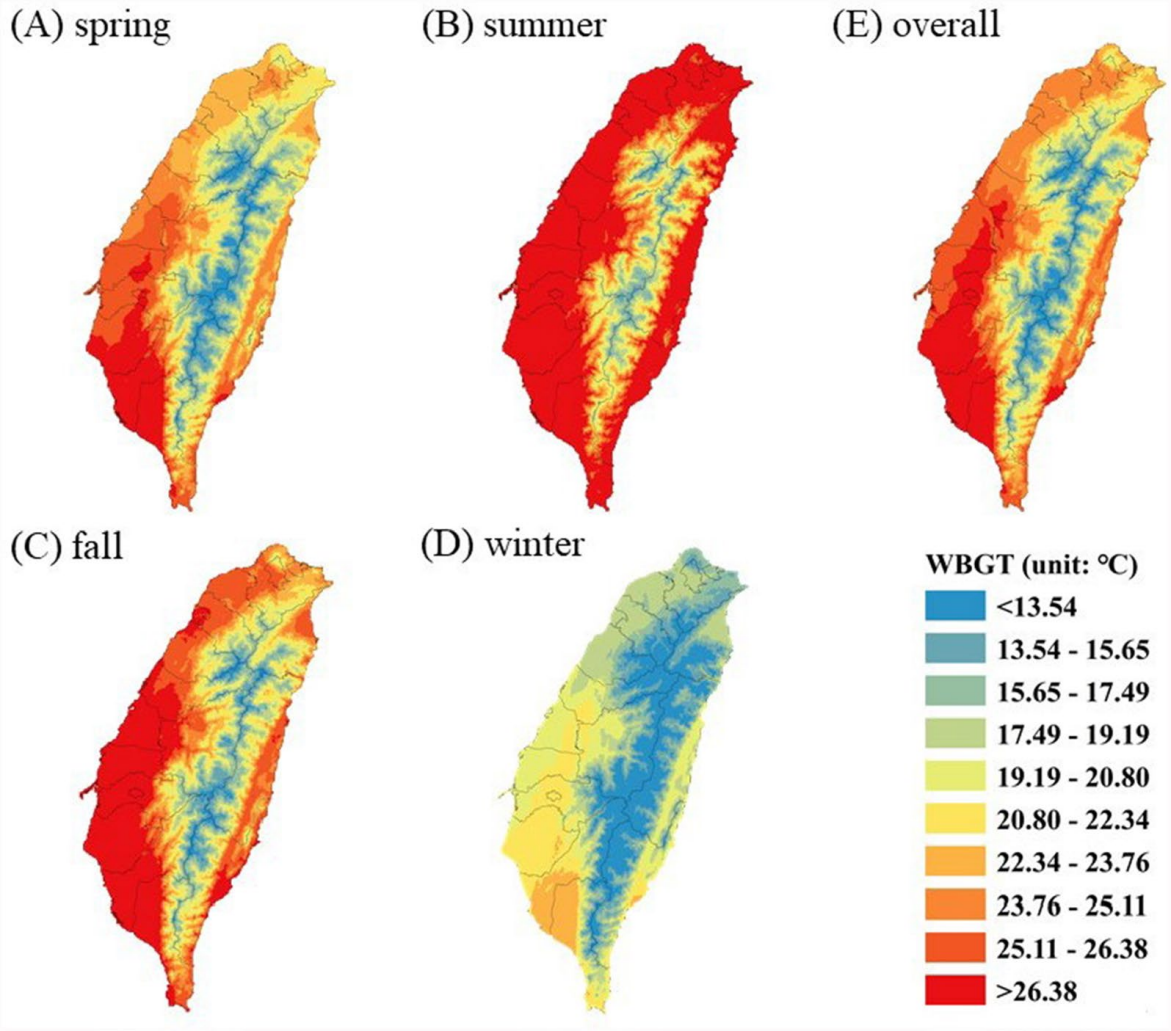
Spatial distribution of WBGT in Taiwan from 2007 to 2017 in seasons. **A** Spring (March to May); **B** summer (June to August); **C** autumn (September to November); **D** winter (December to February);** E** overall

The average WBGT during the study period was 25.0 ± 5.6 °C, ranging from -1.4 to 36.4 °C. Figure 1 illustrates the spatial distribution of WBGT in Taiwan for each season from 2007–2017. The highest average WBGT was recorded in summer (June to August) (26.7 ± 4.8 °C), followed by autumn (September to November) (23.2 ± 5.2 °C), spring (March to May) (21.8 ± 5.7 °C), and winter (December to February) (17.2 ± 5.4 °C).

### Associations between WBGT and epilepsy

**Fig. 2:**
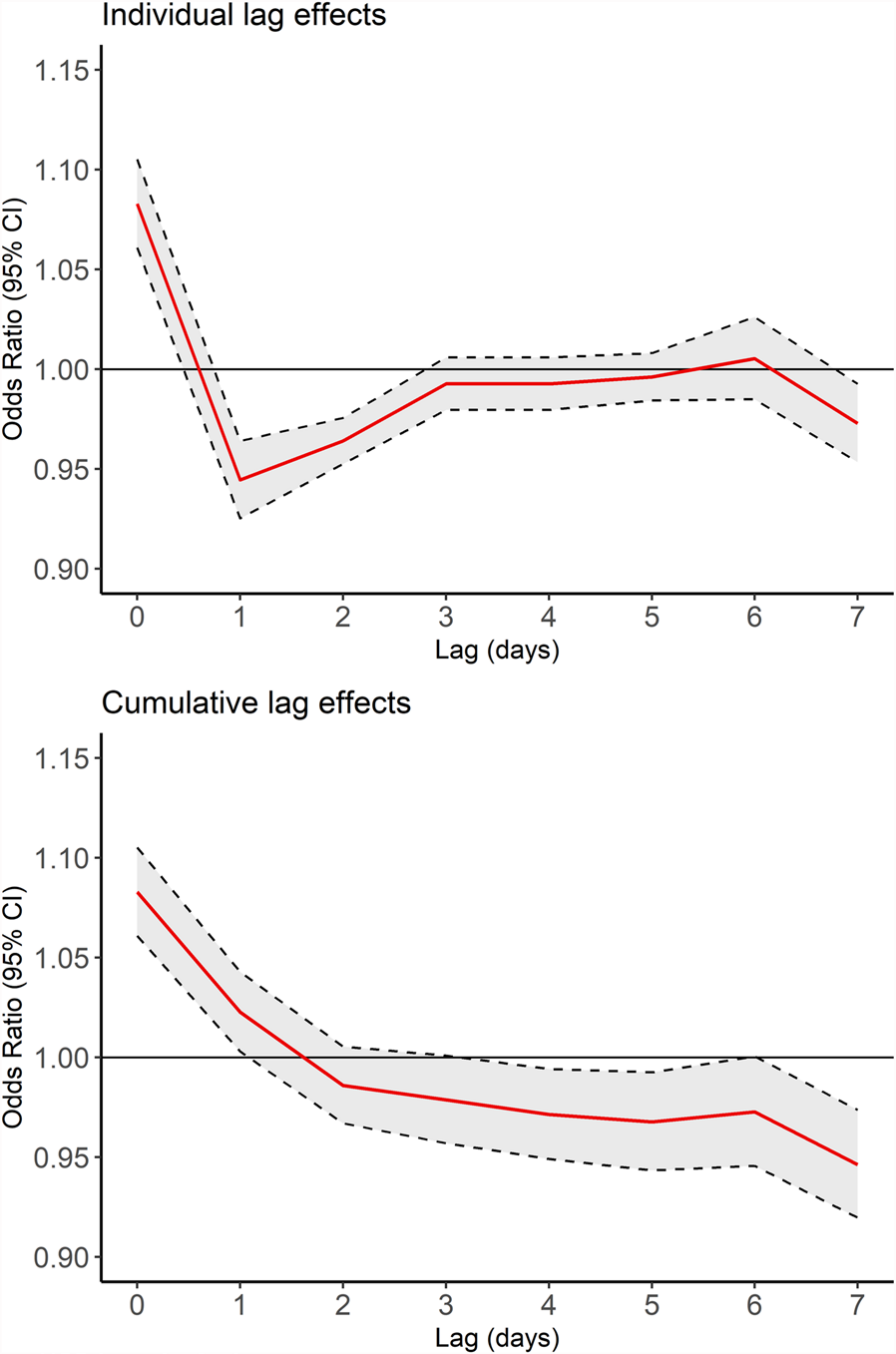
Odds ratio (red line) with 95% confidence interval (CI gray area) of epilepsy with an interquartile range (IQR; 8.9° C) increase in WBGT during the lag 0 to lag 7 by using a distributed lag non-linear model

According to the DLNM model, the lag–response relationship indicated that the associations between an IQR increase in WBGT and epilepsy was significantly positive on the same day (OR = 1.083, 95% CI: 1.061–1.105 at lag 0 day), whereas became significantly negative on the preceding 1–2 days (OR = 0.945, 95% CI 0.925–0.964 at lag 1 day and OR = 0.964, 95% CI 0.953–0.976 at lag 2 day), suggesting a harvesting effect of WBGT on epilepsy (Fig. 2, upper panel). Furthermore, the cumulative effect over lag 0–7 days was significantly negative (OR = 0.946, 95% CI 0.920–0.974) (Fig. 2, lower panel).

**Fig. 3:**
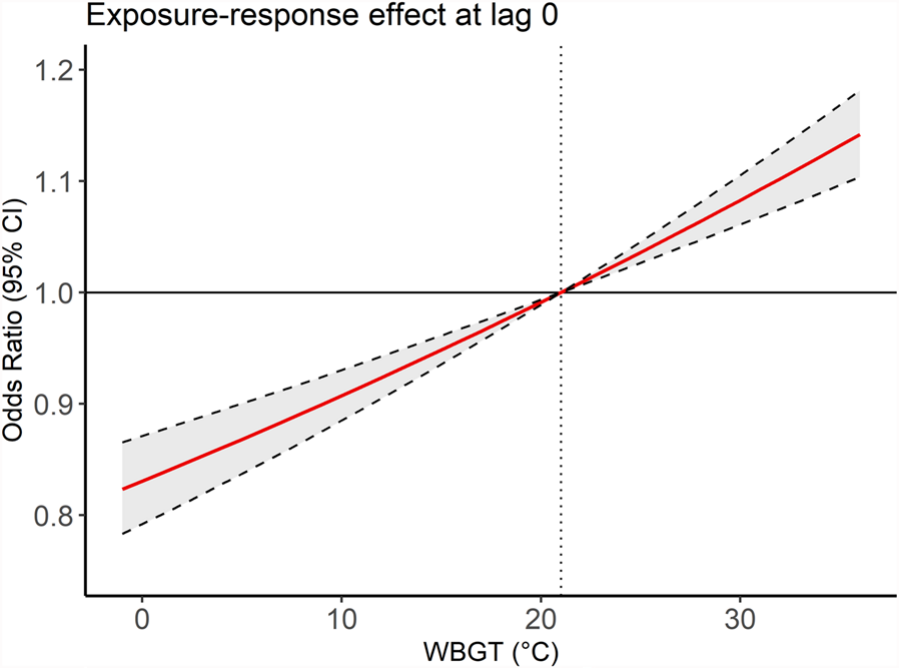
Exposure-response relationship between WBGT exposure and epilepsy at the current day; the red line shows predicted Odds ratio (OR), and the gray area indicates 95% confidence interval (CI) corresponding to WBGT level. The reference value is 21.0 °C

Additionally, the exposure-response relationship between an 8.9℃ increase in WBGT and epilepsy at lag 0 day was linear. No apparent threshold has been observed in the exposure-response relationship between WBGT and epilepsy, suggesting there may be no safe level of WBGT on epilepsy (Fig. 3).

Sensitivity analyses examining different lag periods examining different lag periods and spline types for both exposure-response and lag-response effects are presented in Fig. S1. No substantial differences were observed across the models. A significant positive association between WBGT and epilepsy remained at lag 0 day, along with significant negative associations at lag 1 and lag 2 day (Fig. S1), supporting the robustness of our findings.

Stratified analyses showed that both males and females exhibited significant positive associations at lag 0 day and significant negative associations at lag 1 and lag 2 day (Fig. S2). The exposure-response relationships at lag 0 day are linear in both groups (Fig. S2). Furthermore, the associations between WBGT and epilepsy are stronger in adults (20–39 and 40–64 years) and elderly (≥ 65 years) compared to the children and adolescents group (< 20 years). No significant associations, neither lag-response and nor exposure-response effects, were observed in the children and adolescents group (Fig. S3). In addition, the associations between WBGT and epilepsy appeared to diminish with increasing SES levels. No significant associations were found in the highest SES group (monthly income > $27,600 NTD; Fig. S4).

## Discussion

In this study, we found that WBGT was significantly associated with an increased ED visits for epilepsy and exhibited a linear dose-response relationship. A significant positive association between an 8.9 °C increase in WBGT and epilepsy was observed at lag 0 day, while significant negative associations were observed at lag 1 and lag 2 days (Fig. 2), indicating a harvesting effect of WBGT on epilepsy. No WBGT threshold was identified in the exposure-response relationship (Fig. 3). Additionally, the associations between WBGT and epilepsy were stronger among adults and elders (age ≥ 20 years; Fig. S3) and among individuals with low to medium SES levels (monthly income ≤ $27,600; Fig. S4).

Zhang et al. [13] conducted a time-stratified case-control study to assess the association between heat exposure and epileptic seizures in Brazil. They observed significant positive associations between temperature and epileptic seizure from lag 0 to lag 4 [13]. The overall association between a 1 ℃ increase in daily temperature and epileptic seizure was 1.043 (95% CI 1.028–1.058) [13]. A temperature threshold of 26 ℃ was identified [13]. Additionally, stronger associations were found in the group aged 20–39 years and 40–59 years, whereas no significant associations was observed in the group aged 0–4 and 5–19 years [13]. Fan et al. [14] employed a space-time-stratified case-control study to assess the effects of extreme temperature (at the 97.5th percentile) on childhood epilepsy in Anhui Province, China [14]. They reported significant positive associations between extreme heat and childhood epilepsy at lag 0 (OR = 1.229, 95% CI 1.035–1.459) and lag 1 days (OR = 1.101, 95% CI 1.017–1.191) [14]. Although girls appeared more susceptible to extreme heat than boys, the difference was not statistically significant [14]. Our findings are consistent with those of Zhang et al. [13] and Fan et al. [14], showing that heat exposure (quantified by WBGT) was positively associated with epilepsy risk. The heat effects were generally immediate and occurred within 1 week. Moreover, our results indicated that the associations between heat and epilepsy were stronger in the group aged ≥ 20 years compared to those aged < 20 years, which is consistent with the findings of Zhang et al. [13] in Brazil.

The biological mechanisms underlying the relationship between WBGT and epilepsy risk remain unclear. Several studies have reported that ambient temperature and humidity may be associated with seizure occurrence [2, 3, 6]. Seizure precipitation is expected to increase with rising WBGT levels, considering the combined effects of ambient temperature, humidity, solar radiation, and wind velocity. Precipitants may act directly, influencing human physiology, or indirectly, through socioeconomic disruption such as stress, fatigue, and sleep deprivation, which are well-established seizure triggers [22, 23]. Additionally, the increased frequency of sustained high temperatures and temperature peaks could trigger epilepsy through genetic mechanisms, mediated by genetic variants that modulate physiological responses to heat stress [28].

Studies on human genetics and fever-sensitive epilepsies suggest that genetic polymorphisms that could potentially be associated with seizure susceptibility. Furthermore, exertional heat stress, characterized by an increase in core body temperature due to an imbalance between body heat gain and heat loss, is another cause of hyperthermia [25], which may contribute to seizure onset. Several gene mutations have been associated with temperature-sensitive characteristics, including dysfunctional ion channels, protein expression, and function, which may contribute to epilepsy. Key genes implicated in these mechanisms include SCN1A, SCN8A, GABRG2, and STX1B [26–29]. Some SCN1A mutations linked to epilepsy can lead to channel dysfunction [29]. Dysregulation of body temperature has been reported in DS [30]. Hot-water epilepsy, a form of reflex epilepsy, is characterized by seizures triggered by exposure to hot water, such as bathing or pouring hot water on the head [31]. A Dutch cohort study on DS patients with pathogenic SCN1A mutations found that 41% of seizures were associated with ambient warmth or sudden temperature shifts between cold and warm environments [8]. Empirical and modeling studies demonstrated that elevated temperatures can directly affect the biophysical properties of sodium channels and neuronal dynamics [32]. Similar findings have been observed in severe myoclonic epilepsy in infants, where seizures worsened after exposure to hot water baths [33]. A UK-based study further reported that children experienced more seizures during prolonged periods of atypically high ambient temperatures of summer [8]. The transient receptor potential (TRP) cation channels, which serve as the primary human temperature sensors, are a family of temperature-sensitive ion channels activated in response to specific temperature fluctuations [34]. Findings from the Genetic epilepsy with febrile seizures plus (GEFS +) model suggest that decreased GABAergic inhibition may be a potential mechanism underlying temperature-sensitive seizure phenotype [35]. Understanding theses mechanistic insights could provide a deeper understanding of the relationship between increased body temperature and seizures susceptibility.

Genetic susceptibility increases brain temperature, affecting the permeability and function of native ion channels, such as TRPV channels and L-type Ca^2+^ channels, which influence both excitatory and inhibitory neurons [36, 37]. Activation of the innate immune system during fever and hyperthermia can contribute to seizure precipitation if pro-inflammatory cytokines, such as IL-1β and TNF, exceed their homeostatic threshold [38]. In addition to channel dysfunction, systemic inflammation may also play a role in temperature-related seizures. An increase in core temperature to 40 ± 2 °C for 3–5 min has been shown to elevated blood pressure and induce blood-brain barrier (BBB) breakdown in adult rats [39]. These findings suggest that elevated body temperature may act as a ‘‘second hit” in the control of epileptic seizures and seizure-related brain damage. Furthermore, experimental studies using vertebrate model organisms indicate that heat stress is sufficient to trigger electrographic and behavioral seizures, providing additional experimental evidence that temperature can influence seizure susceptibility in both mature and developing brains [36, 40].

Beyond its physiological effects, extreme heat is projected to increase population stress levels, significantly impacting human psychosocial health. Heat-related stress may pose a serious challenge to seizure control, particularly for individuals with fever-sensitive or stress-sensitive epilepsies [40]. Extreme heat exposure has been associated with a reduced tolerance to high temperatures, which could potentially exacerbate seizure susceptibility in vulnerable populations. A hospital-based bidirectional case-crossover study identified a potential link between higher relative air humidity and an increased risk of epileptic seizures admissions [6]. Additionally, higher ambient temperature was associated with an increased risk of seizure attack [5, 41, 42]. Among the most common reported seizure precipitant, patients frequently recall excess sunlight exposure, fever (3.9%), and abrupt weather changes as significant triggers [43]. Considering the high incidence of febrile seizures in children, which are triggered by hyperthermia-induced respiratory alkalosis and a subsequent increase in neuronal activity [44], we anticipated that if temperature is associated with epileptic seizure risk, particularly warm or hot weather would be a major risk factor. Additionally, elevated body temperature has shown to increases hippocampal neuronal activity, a key mechanism involved in mesial temporal lobe epilepsy [45].

Cerebrovascular diseases, particularly stroke, are important risk factors for both early seizure (ES), defined as seizures occurring concurrently with the ischemic event or within 7 days, and post-stroke epilepsy (PSE), characterized by seizures occurring more than 7 days after the event [46]. Stroke leads to immediate ion channel dysfunction, resulting in increased intracellular sodium and calcium levels, sustained depolarization of the transmembrane potential, and a lowered seizure threshold [46]. A prior review reported that cortical involvement (OR = 3.71, 95% CI 2.34–5.90), cerebral hemorrhage (OR = 2.41, 95% CI 1.57–3.70), and ES (OR = 4.43, 95% CI 2.36–8.32) are associated with an increased risk of PSE [47]. Both low and high temperatures are associated with an increased risk of major adverse cerebrovascular events, and high temperatures are associated with increased risk of ischemic stroke [48]. In our stratified analysis by age group, the associations between WBGT and epilepsy were stronger among adults and elderly than children and adolescents, providing evidence that the association between WBGT and epilepsy may be mediated through cerebrovascular pathways. These findings also suggest that adults and the elderly are more vulnerable to heat-related epilepsy.

The present study has several strengths. First, we used longitudinal national population-based data from the NHIRD, which cover 99.9% of the population in Taiwan. The large database allowed us to collect the largest number of seizure events compared to previous studies, providing sufficient statistical power to assess the association between WBGT and epileptic seizures. Second, we utilized a high 1-km resolution spatiotemporal model, which enhanced the precision of WBGT exposure assessment at the individual level by accounting for spatial and temporal variability [16]. Third, we employed a space-time-stratified case-crossover design, which effectively controlled for potential confounding factors such as sex, age, socioeconomic status, smoking history, occupational history, and genetics, while also minimizing bias related to seasonality and the day of the week (the effects of holidays).

Despite these strengths, our study has several limitations. First, residual confounding factors, such as stress and infection, could not be completely excluded, potentially introduced bias in our findings. Second, we did not have information on participants’ residential movement history and time-activity patterns, which may have led to misclassification bias in WBGT exposure assessment. However, this misclassification is likely non-differential between the case and control periods, minimizing its potential impact on the results.

## Conclusion

In conclusion, we found that heat exposure may trigger the onset of epilepsy on the same day (lag 0), with no apparent threshold identified. Although harvesting effects were observed at lag 1 and 2 days, the significant positive association at lag 0 day suggests that heat exposure may lead to short-term clustering of epilepsy cases. This clustering could still increase the demand for emergency health services, highlighting the need for effective heat management strategies [49].

The impact of heat on epilepsy is multifaceted, occurring not only through direct temperature-related physiological changes but also through the associated psychosocial factors, such as increased stress, sleep deprivation, and fatigue. As precision medicine gains traction, incorporating meteorological variables and heat stress indicators, such as WBGT, into individualized epilepsy management strategies may enhance patient care. Heat may induce stress, fatigue, and sleep deprivation, thereby potentially putting many people with epilepsy at risk of deterioration of seizure control, as well as possible consequences on associated comorbidities and non-seizure aspects of epilepsy. Recognizing these environmental seizure precipitants may aid in developing preventive strategies for epilepsy.

## Supplementary Information

Supplementary material 1.

## Data Availability

The data that support the findings of this study are available from the Health and Welfare Data Center (HWDC), Taiwan’s Ministry of Health and Welfare (MOHW) but restrictions apply to the availability of these data, which were used under license for the current study. Data are however available from authors upon reasonable request and with permission of HWDC, MOHW.

## Abbreviations

AIC: Akaike information criterion
CI: Confidence interval
DF: Degree of freedom
DLNM: Distributed lag non-linear model
DS: Dravet syndrome
ECMWF: European Centre for Medium-Range Weather Forecasts
ED: Emergency department
ES: Early seizures
ICD: International Classification of Diseases
IQR: Interquartile range
MOHW: Ministry of Health and Welfare
NHIRD: National Health Insurance Research Database
OR: Odds ratio
PSE: Post-stroke epilepsy
SES: Socioeconomic status
TCCIP: Taiwan Climate Change Projection and Information Platform
TRP: Transient receptor potential
WBGT: Wet-bulb globe temperature
