# Supplementary material for: Association between wet-bulb globe temperature and epilepsy: a space-time-stratified case-crossover study in Taiwan
Source: Trop Med Health. 2025 May 20;53:72. doi: 10.1186/s41182-025-00755-z (PMC12090581; doi:10.1186/s41182-025-00755-z)
Supplement: Supplementary file 1 — Supplementary material 1. [file 41182_2025_755_MOESM1_ESM.docx]

**Supplementary material**

**Association between wet-bulb globe temperature and** **epilepsy: a space-time-stratified case-crossover study in Taiwan**

Yu-Tzu Chang, Yu-Ting Lin, Bao-Ru Chuang, Wen-Hsuan Chuang, Bing-Fang Hwang, Chau-Ren Jung

**Table of content**

**Table S1.** Summary of previous studies assessing the associations between meteorological factors and seizure occurrence.

**Table S2**. Comparison of Akaike Information Criterion (AIC) across various combinations of lag periods, spline types, and the degree of freedom (df) for modeling lag-response and exposure-response relationships. The lowest AIC value is highlighted in green color.

**Fig. S1**. Sensitivity analyses examining different lag periods (0–7, 0–10, and 0–21 lag days) and spline types, including a B-spline (bs) with 4 degree of freedom (df) for the exposure-response effect and a B-spline with 5 df for the lag-response effect. Shaded grey areas represent the 95% confidence intervals.

**Fig. S2.** Stratified analysis by sex. (Left panel) individual lag effects of wet-bulb globe temperature (WBGT) on epilepsy; (right panel) exposure-response relationships.

**Fig. S3.** Stratified analysis by age group. (Left panel) individual lag effects of wet-bulb globe temperature (WBGT) on epilepsy; (right panel) exposure-response relationships.

**Fig. S4.** Stratified analysis by socioeconomic status. (Left panel) individual lag effects of wet-bulb globe temperature (WBGT) on epilepsy; (right panel) exposure-response relationships.

**Table S1.** Summary of previous studies assessing the associations between meteorological factors and seizure occurrence.

| **Author, Year** | **Country (region)** | **Meteorological factors** | | | | | | **Descriptions** |
| --- | --- | --- | --- | --- | --- | --- | --- | --- |
|  |  | **Temperature** | **Relative humidity** | **Atmospheric pressure** | **Sunshine duration** | **Precipitation** | **Wind velocity** |  |
| Rüegg et al., 2008 | Switzerland | Negative | Negative | - | - | - | No association |  |
| Rakers et al., 2017 | Jena, Central Germany | Negative | Positive | Positive | - | - | - |  |
| Brás et al., 2018 | Lisbon, Portugal | Negative | Positive | Positive | Negative |  |  |  |
| Chang et al, 2019 | Taiwan | Negative | No association | No association | No association | Negative | - | In multivariate analysis, on the association of temperature remains significant |
| Zhang et al., 2023 | Brazil | Positive | - | - | - | - | - |  |
| Fang et al., 2024 | Anhui, China | A U-shape, both heat and cold was positively correlated with epilepsy | - | - | - | - | - | Outcome was children epilepsy hospitalization |
| Hammen et al., 2024 | Germany | Negative | Positive | Positive |  | Negative for generalized epilepsy, while positive for seizures with unknown onset and unclassified seizures |  | Negative associations between temperature and seizure was observed for unclassified seizures, while positive associations were found for seizures with unknown onset |

**Table S2**. Comparison of Akaike Information Criterion (AIC) across various combinations of lag periods, spline types, and the degree of freedom (df) for modeling lag-response and exposure-response relationships. The lowest AIC value is highlighted in green color.

| **Lag periods** | **Types of exposure-response effect** | **degree of freedom** | **Type of lag-response effect** | **degree of freedom** | **AIC** |
| --- | --- | --- | --- | --- | --- |
| lag 0–5 | B-spline | 4 | B-spline | 4 | 553403.5 |
| lag 0–5 | B-spline | 4 | B-spline | 5 | 553401.2 |
| lag 0–5 | B-spline | 5 | B-spline | 4 | 553407.5 |
| lag 0–5 | B-spline | 5 | B-spline | 5 | 553406.4 |
| lag 0–5 | B-spline | 4 | Natural cubic spline | 3 | 553429.7 |
| lag 0–5 | B-spline | 4 | Natural cubic spline | 4 | 553404.2 |
| lag 0–5 | B-spline | 4 | Natural cubic spline | 5 | 553400.9 |
| lag 0–5 | B-spline | 5 | Natural cubic spline | 3 | 553431.4 |
| lag 0–5 | B-spline | 5 | Natural cubic spline | 4 | 553408.2 |
| lag 0–5 | B-spline | 5 | Natural cubic spline | 5 | 553406.2 |
| lag 0–5 | Natural cubic spline | 3 | Natural cubic spline | 3 | 553426.1 |
| lag 0–5 | Natural cubic spline | 3 | Natural cubic spline | 4 | 553400.9 |
| lag 0–5 | Natural cubic spline | 3 | Natural cubic spline | 5 | 553397.0 |
| lag 0–5 | Natural cubic spline | 4 | Natural cubic spline | 3 | 553429.3 |
| lag 0–5 | Natural cubic spline | 4 | Natural cubic spline | 4 | 553404.0 |
| lag 0–5 | Natural cubic spline | 4 | Natural cubic spline | 5 | 553401.9 |
| lag 0–5 | Natural cubic spline | 5 | Natural cubic spline | 3 | 553433.3 |
| lag 0–5 | Natural cubic spline | 5 | Natural cubic spline | 4 | 553410.0 |
| lag 0–5 | Natural cubic spline | 5 | Natural cubic spline | 5 | 553409.4 |
| lag 0–5 | Natural cubic spline | 3 | B-spline | 4 | 553400.0 |
| lag 0–5 | Natural cubic spline | 3 | B-spline | 5 | 553397.1 |
| lag 0–5 | Natural cubic spline | 4 | B-spline | 4 | 553403.9 |
| lag 0–5 | Natural cubic spline | 4 | B-spline | 5 | 553402.4 |
| lag 0–5 | Natural cubic spline | 5 | B-spline | 4 | 553409.8 |
| lag 0–5 | Natural cubic spline | 5 | B-spline | 5 | 553409.9 |
| lag 0–5 | Linear | - | B-spline | 4 | 553390.2 |
| lag 0–5 | Linear | - | B-spline | 5 | 553385.7 |
| lag 0–5 | Linear | - | B-spline | 6 | 553387.6 |
| lag 0–5 | Linear | - | B-spline | 7 | 553387.6 |
| lag 0–5 | Linear | - | Natural cubic spline | 3 | 553419.7 |
| lag 0–5 | Linear | - | Natural cubic spline | 4 | 553391.4 |
| lag 0–5 | Linear | - | Natural cubic spline | 5 | 553385.6 |
| lag 0–5 | Linear | - | Natural cubic spline | 6 | 553387.6 |
| lag 0–5 | Linear | - | Natural cubic spline | 7 | 553387.6 |
| lag 0–5 | B-spline | 4 | Linear | - | 553463.6 |
| lag 0–5 | B-spline | 5 | Linear | - | 553464.8 |
| lag 0–5 | B-spline | 6 | Linear | - | 553465.3 |
| lag 0–5 | B-spline | 7 | Linear | - | 553469.1 |
| lag 0–5 | Natural cubic spline | 3 | Linear | - | 553461.3 |
| lag 0–5 | Natural cubic spline | 4 | Linear | - | 553462.6 |
| lag 0–5 | Natural cubic spline | 5 | Linear | - | 553466.0 |
| lag 0–5 | Natural cubic spline | 6 | Linear | - | 553466.8 |
| lag 0–5 | Natural cubic spline | 7 | Linear | - | 553463.1 |
| lag 0–5 | Linear | - | Linear | - | 553457.8 |
| lag 0–6 | B-spline | 4 | B-spline | 4 | 553404.0 |
| lag 0–6 | B-spline | 4 | B-spline | 5 | 553401.5 |
| lag 0–6 | B-spline | 5 | B-spline | 4 | 553405.5 |
| lag 0–6 | B-spline | 5 | B-spline | 5 | 553405.1 |
| lag 0–6 | B-spline | 4 | Natural cubic spline | 3 | 553445.6 |
| lag 0–6 | B-spline | 4 | Natural cubic spline | 4 | 553407.8 |
| lag 0–6 | B-spline | 4 | Natural cubic spline | 5 | 553402.4 |
| lag 0–6 | B-spline | 5 | Natural cubic spline | 3 | 553445.1 |
| lag 0–6 | B-spline | 5 | Natural cubic spline | 4 | 553409.2 |
| lag 0–6 | B-spline | 5 | Natural cubic spline | 5 | 553406.0 |
| lag 0–6 | Natural cubic spline | 3 | Natural cubic spline | 3 | 553443.4 |
| lag 0–6 | Natural cubic spline | 3 | Natural cubic spline | 4 | 553406.1 |
| lag 0–6 | Natural cubic spline | 3 | Natural cubic spline | 5 | 553398.7 |
| lag 0–6 | Natural cubic spline | 4 | Natural cubic spline | 3 | 553447.0 |
| lag 0–6 | Natural cubic spline | 4 | Natural cubic spline | 4 | 553409.4 |
| lag 0–6 | Natural cubic spline | 4 | Natural cubic spline | 5 | 553403.9 |
| lag 0–6 | Natural cubic spline | 5 | Natural cubic spline | 3 | 553450.0 |
| lag 0–6 | Natural cubic spline | 5 | Natural cubic spline | 4 | 553414.0 |
| lag 0–6 | Natural cubic spline | 5 | Natural cubic spline | 5 | 553410.7 |
| lag 0–6 | Natural cubic spline | 3 | B-spline | 4 | 553401.9 |
| lag 0–6 | Natural cubic spline | 3 | B-spline | 5 | 553397.5 |
| lag 0–6 | Natural cubic spline | 4 | B-spline | 4 | 553405.8 |
| lag 0–6 | Natural cubic spline | 4 | B-spline | 5 | 553403.2 |
| lag 0–6 | Natural cubic spline | 5 | B-spline | 4 | 553410.4 |
| lag 0–6 | Natural cubic spline | 5 | B-spline | 5 | 553409.9 |
| lag 0–6 | Linear | - | B-spline | 4 | 553392.8 |
| lag 0–6 | Linear | - | B-spline | 5 | 553385.9 |
| lag 0–6 | Linear | - | B-spline | 6 | 553385.7 |
| lag 0–6 | Linear | - | B-spline | 7 | 553386.7 |
| lag 0–6 | Linear | - | Natural cubic spline | 3 | 553435.7 |
| lag 0–6 | Linear | - | Natural cubic spline | 4 | 553397.6 |
| lag 0–6 | Linear | - | Natural cubic spline | 5 | 553387.6 |
| lag 0–6 | Linear | - | Natural cubic spline | 6 | 553385.1 |
| lag 0–6 | Linear | - | Natural cubic spline | 7 | 553386.7 |
| lag 0–6 | B-spline | 4 | Linear | - | 553468.2 |
| lag 0–6 | B-spline | 5 | Linear | - | 553468.0 |
| lag 0–6 | B-spline | 6 | Linear | - | 553468.8 |
| lag 0–6 | B-spline | 7 | Linear | - | 553472.0 |
| lag 0–6 | Natural cubic spline | 3 | Linear | - | 553463.7 |
| lag 0–6 | Natural cubic spline | 4 | Linear | - | 553466.8 |
| lag 0–6 | Natural cubic spline | 5 | Linear | - | 553470.4 |
| lag 0–6 | Natural cubic spline | 6 | Linear | - | 553470.4 |
| lag 0–6 | Natural cubic spline | 7 | Linear | - | 553467.2 |
| lag 0–6 | Linear | - | Linear | - | 553459.1 |
| lag 0–7 | B-spline | 4 | B-spline | 4 | 553403.1 |
| lag 0–7 | B-spline | 4 | B-spline | 5 | 553397.8 |
| lag 0–7 | B-spline | 5 | B-spline | 4 | 553404.7 |
| lag 0–7 | B-spline | 5 | B-spline | 5 | 553400.9 |
| lag 0–7 | B-spline | 4 | Natural cubic spline | 3 | 553450.7 |
| lag 0–7 | B-spline | 4 | Natural cubic spline | 4 | 553409.4 |
| lag 0–7 | B-spline | 4 | Natural cubic spline | 5 | 553402.0 |
| lag 0–7 | B-spline | 5 | Natural cubic spline | 3 | 553451.9 |
| lag 0–7 | B-spline | 5 | Natural cubic spline | 4 | 553410.7 |
| lag 0–7 | B-spline | 5 | Natural cubic spline | 5 | 553405.4 |
| lag 0–7 | Natural cubic spline | 3 | Natural cubic spline | 3 | 553453.0 |
| lag 0–7 | Natural cubic spline | 3 | Natural cubic spline | 4 | 553410.8 |
| lag 0–7 | Natural cubic spline | 3 | Natural cubic spline | 5 | 553402.3 |
| lag 0–7 | Natural cubic spline | 4 | Natural cubic spline | 3 | 553453.4 |
| lag 0–7 | Natural cubic spline | 4 | Natural cubic spline | 4 | 553411.4 |
| lag 0–7 | Natural cubic spline | 4 | Natural cubic spline | 5 | 553404.3 |
| lag 0–7 | Natural cubic spline | 5 | Natural cubic spline | 3 | 553458.1 |
| lag 0–7 | Natural cubic spline | 5 | Natural cubic spline | 4 | 553416.8 |
| lag 0–7 | Natural cubic spline | 5 | Natural cubic spline | 5 | 553411.2 |
| lag 0–7 | Natural cubic spline | 3 | B-spline | 4 | 553404.5 |
| lag 0–7 | Natural cubic spline | 3 | B-spline | 5 | 553398.0 |
| lag 0–7 | Natural cubic spline | 4 | B-spline | 4 | 553405.3 |
| lag 0–7 | Natural cubic spline | 4 | B-spline | 5 | 553400.5 |
| lag 0–7 | Natural cubic spline | 5 | B-spline | 4 | 553410.5 |
| lag 0–7 | Natural cubic spline | 5 | B-spline | 5 | 553407.2 |
| lag 0–7 | Linear | - | B-spline | 4 | 553392.8 |
| lag 0–7 | Linear | - | B-spline | 5 | 553383.7 |
| **lag 0–7** | **Linear** | **-** | **B-spline** | **6** | **553379.4** |
| lag 0–7 | Linear | - | B-spline | 7 | 553381.8 |
| lag 0–7 | Linear | - | Natural cubic spline | 3 | 553445.1 |
| lag 0–7 | Linear | - | Natural cubic spline | 4 | 553399.7 |
| lag 0–7 | Linear | - | Natural cubic spline | 5 | 553388.6 |
| lag 0–7 | Linear | - | Natural cubic spline | 6 | 553379.7 |
| lag 0–7 | Linear | - | Natural cubic spline | 7 | 553381.1 |
| lag 0–7 | B-spline | 4 | Linear | - | 553462.5 |
| lag 0–7 | B-spline | 5 | Linear | - | 553462.3 |
| lag 0–7 | B-spline | 6 | Linear | - | 553462.9 |
| lag 0–7 | B-spline | 7 | Linear | - | 553465.0 |
| lag 0–7 | Natural cubic spline | 3 | Linear | - | 553458.5 |
| lag 0–7 | Natural cubic spline | 4 | Linear | - | 553462.3 |
| lag 0–7 | Natural cubic spline | 5 | Linear | - | 553465.9 |
| lag 0–7 | Natural cubic spline | 6 | Linear | - | 553465.8 |
| lag 0–7 | Natural cubic spline | 7 | Linear | - | 553461.7 |
| lag 0–7 | Linear | - | Linear | - | 553453.5 |
| lag 0–8 | B-spline | 4 | B-spline | 4 | 553417.6 |
| lag 0–8 | B-spline | 4 | B-spline | 5 | 553393.9 |
| lag 0–8 | B-spline | 5 | B-spline | 4 | 553418.2 |
| lag 0–8 | B-spline | 5 | B-spline | 5 | 553396.1 |
| lag 0–8 | B-spline | 4 | Natural cubic spline | 3 | 553449.0 |
| lag 0–8 | B-spline | 4 | Natural cubic spline | 4 | 553424.1 |
| lag 0–8 | B-spline | 4 | Natural cubic spline | 5 | 553400.6 |
| lag 0–8 | B-spline | 5 | Natural cubic spline | 3 | 553451.9 |
| lag 0–8 | B-spline | 5 | Natural cubic spline | 4 | 553424.5 |
| lag 0–8 | B-spline | 5 | Natural cubic spline | 5 | 553402.6 |
| lag 0–8 | Natural cubic spline | 3 | Natural cubic spline | 3 | 553454.0 |
| lag 0–8 | Natural cubic spline | 3 | Natural cubic spline | 4 | 553427.3 |
| lag 0–8 | Natural cubic spline | 3 | Natural cubic spline | 5 | 553404.1 |
| lag 0–8 | Natural cubic spline | 4 | Natural cubic spline | 3 | 553450.9 |
| lag 0–8 | Natural cubic spline | 4 | Natural cubic spline | 4 | 553425.6 |
| lag 0–8 | Natural cubic spline | 4 | Natural cubic spline | 5 | 553403.1 |
| lag 0–8 | Natural cubic spline | 5 | Natural cubic spline | 3 | 553456.3 |
| lag 0–8 | Natural cubic spline | 5 | Natural cubic spline | 4 | 553430.9 |
| lag 0–8 | Natural cubic spline | 5 | Natural cubic spline | 5 | 553409.9 |
| lag 0–8 | Natural cubic spline | 3 | B-spline | 4 | 553420.5 |
| lag 0–8 | Natural cubic spline | 3 | B-spline | 5 | 553397.0 |
| lag 0–8 | Natural cubic spline | 4 | B-spline | 4 | 553419.0 |
| lag 0–8 | Natural cubic spline | 4 | B-spline | 5 | 553396.2 |
| lag 0–8 | Natural cubic spline | 5 | B-spline | 4 | 553424.5 |
| lag 0–8 | Natural cubic spline | 5 | B-spline | 5 | 553403.4 |
| lag 0–8 | Linear | - | B-spline | 4 | 553410.9 |
| lag 0–8 | Linear | - | B-spline | 5 | 553384.9 |
| lag 0–8 | Linear | - | B-spline | 6 | 553379.6 |
| lag 0–8 | Linear | - | B-spline | 7 | 553379.4 |
| lag 0–8 | Linear | - | Natural cubic spline | 3 | 553447.7 |
| lag 0–8 | Linear | - | Natural cubic spline | 4 | 553417.4 |
| lag 0–8 | Linear | - | Natural cubic spline | 5 | 553393.2 |
| lag 0–8 | Linear | - | Natural cubic spline | 6 | 553384.5 |
| lag 0–8 | Linear | - | Natural cubic spline | 7 | 553379.0 |
| lag 0–8 | B-spline | 4 | Linear | - | 553462.6 |
| lag 0–8 | B-spline | 5 | Linear | - | 553463.3 |
| lag 0–8 | B-spline | 6 | Linear | - | 553464.3 |
| lag 0–8 | B-spline | 7 | Linear | - | 553466.7 |
| lag 0–8 | Natural cubic spline | 3 | Linear | - | 553461.1 |
| lag 0–8 | Natural cubic spline | 4 | Linear | - | 553463.9 |
| lag 0–8 | Natural cubic spline | 5 | Linear | - | 553467.4 |
| lag 0–8 | Natural cubic spline | 6 | Linear | - | 553467.5 |
| lag 0–8 | Natural cubic spline | 7 | Linear | - | 553464.7 |
| lag 0–8 | Linear | - | Linear | - | 553455.9 |
| lag 0–9 | B-spline | 4 | B-spline | 4 | 553436.7 |
| lag 0–9 | B-spline | 4 | B-spline | 5 | 553414.7 |
| lag 0–9 | B-spline | 5 | B-spline | 4 | 553438.2 |
| lag 0–9 | B-spline | 5 | B-spline | 5 | 553417.4 |
| lag 0–9 | B-spline | 4 | Natural cubic spline | 3 | 553454.9 |
| lag 0–9 | B-spline | 4 | Natural cubic spline | 4 | 553445.8 |
| lag 0–9 | B-spline | 4 | Natural cubic spline | 5 | 553424.1 |
| lag 0–9 | B-spline | 5 | Natural cubic spline | 3 | 553454.7 |
| lag 0–9 | B-spline | 5 | Natural cubic spline | 4 | 553447.3 |
| lag 0–9 | B-spline | 5 | Natural cubic spline | 5 | 553425.9 |
| lag 0–9 | Natural cubic spline | 3 | Natural cubic spline | 3 | 553458.6 |
| lag 0–9 | Natural cubic spline | 3 | Natural cubic spline | 4 | 553450.7 |
| lag 0–9 | Natural cubic spline | 3 | Natural cubic spline | 5 | 553427.1 |
| lag 0–9 | Natural cubic spline | 4 | Natural cubic spline | 3 | 553459.0 |
| lag 0–9 | Natural cubic spline | 4 | Natural cubic spline | 4 | 553451.2 |
| lag 0–9 | Natural cubic spline | 4 | Natural cubic spline | 5 | 553427.9 |
| lag 0–9 | Natural cubic spline | 5 | Natural cubic spline | 3 | 553462.9 |
| lag 0–9 | Natural cubic spline | 5 | Natural cubic spline | 4 | 553456.4 |
| lag 0–9 | Natural cubic spline | 5 | Natural cubic spline | 5 | 553434.7 |
| lag 0–9 | Natural cubic spline | 3 | B-spline | 4 | 553440.8 |
| lag 0–9 | Natural cubic spline | 3 | B-spline | 5 | 553416.7 |
| lag 0–9 | Natural cubic spline | 4 | B-spline | 4 | 553442.1 |
| lag 0–9 | Natural cubic spline | 4 | B-spline | 5 | 553418.6 |
| lag 0–9 | Natural cubic spline | 5 | B-spline | 4 | 553447.1 |
| lag 0–9 | Natural cubic spline | 5 | B-spline | 5 | 553425.7 |
| lag 0–9 | Linear | - | B-spline | 4 | 553430.0 |
| lag 0–9 | Linear | - | B-spline | 5 | 553402.1 |
| lag 0–9 | Linear | - | B-spline | 6 | 553382.8 |
| lag 0–9 | Linear | - | B-spline | 7 | 553381.5 |
| lag 0–9 | Linear | - | Natural cubic spline | 3 | 553451.8 |
| lag 0–9 | Linear | - | Natural cubic spline | 4 | 553440.0 |
| lag 0–9 | Linear | - | Natural cubic spline | 5 | 553412.6 |
| lag 0–9 | Linear | - | Natural cubic spline | 6 | 553394.4 |
| lag 0–9 | Linear | - | Natural cubic spline | 7 | 553388.8 |
| lag 0–9 | B-spline | 4 | Linear | - | 553466.7 |
| lag 0–9 | B-spline | 5 | Linear | - | 553464.2 |
| lag 0–9 | B-spline | 6 | Linear | - | 553464.4 |
| lag 0–9 | B-spline | 7 | Linear | - | 553466.3 |
| lag 0–9 | Natural cubic spline | 3 | Linear | - | 553469.1 |
| lag 0–9 | Natural cubic spline | 4 | Linear | - | 553470.9 |
| lag 0–9 | Natural cubic spline | 5 | Linear | - | 553473.0 |
| lag 0–9 | Natural cubic spline | 6 | Linear | - | 553471.9 |
| lag 0–9 | Natural cubic spline | 7 | Linear | - | 553464.8 |
| lag 0–9 | Linear | - | Linear | - | 553463.5 |
| lag 0–10 | B-spline | 4 | B-spline | 4 | 553436.7 |
| lag 0–10 | B-spline | 4 | B-spline | 5 | 553414.7 |
| lag 0–10 | B-spline | 5 | B-spline | 4 | 553438.2 |
| lag 0–10 | B-spline | 5 | B-spline | 5 | 553417.4 |
| lag 0–10 | B-spline | 4 | Natural cubic spline | 3 | 553454.9 |
| lag 0–10 | B-spline | 4 | Natural cubic spline | 4 | 553445.8 |
| lag 0–10 | B-spline | 4 | Natural cubic spline | 5 | 553424.1 |
| lag 0–10 | B-spline | 5 | Natural cubic spline | 3 | 553454.7 |
| lag 0–10 | B-spline | 5 | Natural cubic spline | 4 | 553447.3 |
| lag 0–10 | B-spline | 5 | Natural cubic spline | 5 | 553425.9 |
| lag 0–10 | Natural cubic spline | 3 | Natural cubic spline | 3 | 553458.6 |
| lag 0–10 | Natural cubic spline | 3 | Natural cubic spline | 4 | 553450.7 |
| lag 0–10 | Natural cubic spline | 3 | Natural cubic spline | 5 | 553427.1 |
| lag 0–10 | Natural cubic spline | 4 | Natural cubic spline | 3 | 553459.0 |
| lag 0–10 | Natural cubic spline | 4 | Natural cubic spline | 4 | 553451.2 |
| lag 0–10 | Natural cubic spline | 4 | Natural cubic spline | 5 | 553427.9 |
| lag 0–10 | Natural cubic spline | 5 | Natural cubic spline | 3 | 553462.9 |
| lag 0–10 | Natural cubic spline | 5 | Natural cubic spline | 4 | 553456.4 |
| lag 0–10 | Natural cubic spline | 5 | Natural cubic spline | 5 | 553434.7 |
| lag 0–10 | Natural cubic spline | 3 | B-spline | 4 | 553440.8 |
| lag 0–10 | Natural cubic spline | 3 | B-spline | 5 | 553416.7 |
| lag 0–10 | Natural cubic spline | 4 | B-spline | 4 | 553442.1 |
| lag 0–10 | Natural cubic spline | 4 | B-spline | 5 | 553418.6 |
| lag 0–10 | Natural cubic spline | 5 | B-spline | 4 | 553447.1 |
| lag 0–10 | Natural cubic spline | 5 | B-spline | 5 | 553425.7 |
| lag 0–10 | Linear | - | B-spline | 4 | 553430.0 |
| lag 0–10 | Linear | - | B-spline | 5 | 553402.1 |
| lag 0–10 | Linear | - | B-spline | 6 | 553382.8 |
| lag 0–10 | Linear | - | B-spline | 7 | 553381.5 |
| lag 0–10 | Linear | - | Natural cubic spline | 3 | 553451.8 |
| lag 0–10 | Linear | - | Natural cubic spline | 4 | 553440.0 |
| lag 0–10 | Linear | - | Natural cubic spline | 5 | 553412.6 |
| lag 0–10 | Linear | - | Natural cubic spline | 6 | 553394.4 |
| lag 0–10 | Linear | - | Natural cubic spline | 7 | 553388.8 |
| lag 0–10 | B-spline | 4 | Linear | - | 553466.7 |
| lag 0–10 | B-spline | 5 | Linear | - | 553464.2 |
| lag 0–10 | B-spline | 6 | Linear | - | 553464.4 |
| lag 0–10 | B-spline | 7 | Linear | - | 553466.3 |
| lag 0–10 | Natural cubic spline | 3 | Linear | - | 553469.1 |
| lag 0–10 | Natural cubic spline | 4 | Linear | - | 553470.9 |
| lag 0–10 | Natural cubic spline | 5 | Linear | - | 553473.0 |
| lag 0–10 | Natural cubic spline | 6 | Linear | - | 553471.9 |
| lag 0–10 | Natural cubic spline | 7 | Linear | - | 553464.8 |
| lag 0–10 | Linear | - | Linear | - | 553463.5 |
| lag 0–21 | B-spline | 4 | B-spline | 4 | 553434.0 |
| lag 0–21 | B-spline | 4 | B-spline | 5 | 553433.0 |
| lag 0–21 | B-spline | 5 | B-spline | 4 | 553432.2 |
| lag 0–21 | B-spline | 5 | B-spline | 5 | 553428.0 |
| lag 0–21 | B-spline | 4 | Natural cubic spline | 3 | 553454.0 |
| lag 0–21 | B-spline | 4 | Natural cubic spline | 4 | 553442.2 |
| lag 0–21 | B-spline | 4 | Natural cubic spline | 5 | 553440.1 |
| lag 0–21 | B-spline | 5 | Natural cubic spline | 3 | 553449.0 |
| lag 0–21 | B-spline | 5 | Natural cubic spline | 4 | 553438.4 |
| lag 0–21 | B-spline | 5 | Natural cubic spline | 5 | 553430.5 |
| lag 0–21 | Natural cubic spline | 3 | Natural cubic spline | 3 | 553470.1 |
| lag 0–21 | Natural cubic spline | 3 | Natural cubic spline | 4 | 553461.8 |
| lag 0–21 | Natural cubic spline | 3 | Natural cubic spline | 5 | 553460.5 |
| lag 0–21 | Natural cubic spline | 4 | Natural cubic spline | 3 | 553473.4 |
| lag 0–21 | Natural cubic spline | 4 | Natural cubic spline | 4 | 553466.0 |
| lag 0–21 | Natural cubic spline | 4 | Natural cubic spline | 5 | 553467.3 |
| lag 0–21 | Natural cubic spline | 5 | Natural cubic spline | 3 | 553472.4 |
| lag 0–21 | Natural cubic spline | 5 | Natural cubic spline | 4 | 553465.0 |
| lag 0–21 | Natural cubic spline | 5 | Natural cubic spline | 5 | 553461.7 |
| lag 0–21 | Natural cubic spline | 3 | B-spline | 4 | 553454.3 |
| lag 0–21 | Natural cubic spline | 3 | B-spline | 5 | 553452.3 |
| lag 0–21 | Natural cubic spline | 4 | B-spline | 4 | 553458.3 |
| lag 0–21 | Natural cubic spline | 4 | B-spline | 5 | 553459.0 |
| lag 0–21 | Natural cubic spline | 5 | B-spline | 4 | 553455.5 |
| lag 0–21 | Natural cubic spline | 5 | B-spline | 5 | 553452.2 |
| lag 0–21 | Linear | - | B-spline | 4 | 553451.5 |
| lag 0–21 | Linear | - | B-spline | 5 | 553449.8 |
| lag 0–21 | Linear | - | B-spline | 6 | 553430.2 |
| lag 0–21 | Linear | - | B-spline | 7 | 553416.0 |
| lag 0–21 | Linear | - | Natural cubic spline | 3 | 553467.5 |
| lag 0–21 | Linear | - | Natural cubic spline | 4 | 553459.0 |
| lag 0–21 | Linear | - | Natural cubic spline | 5 | 553457.5 |
| lag 0–21 | Linear | - | Natural cubic spline | 6 | 553444.2 |
| lag 0–21 | Linear | - | Natural cubic spline | 7 | 553441.0 |
| lag 0–21 | B-spline | 4 | Linear | - | 553462.9 |
| lag 0–21 | B-spline | 5 | Linear | - | 553464.5 |
| lag 0–21 | B-spline | 6 | Linear | - | 553458.1 |
| lag 0–21 | B-spline | 7 | Linear | - | 553458.6 |
| lag 0–21 | Natural cubic spline | 3 | Linear | - | 553467.4 |
| lag 0–21 | Natural cubic spline | 4 | Linear | - | 553470.7 |
| lag 0–21 | Natural cubic spline | 5 | Linear | - | 553473.5 |
| lag 0–21 | Natural cubic spline | 6 | Linear | - | 553472.6 |
| lag 0–21 | Natural cubic spline | 7 | Linear | - | 553470.7 |
| lag 0–21 | Linear | - | Linear | - | 553465.6 |


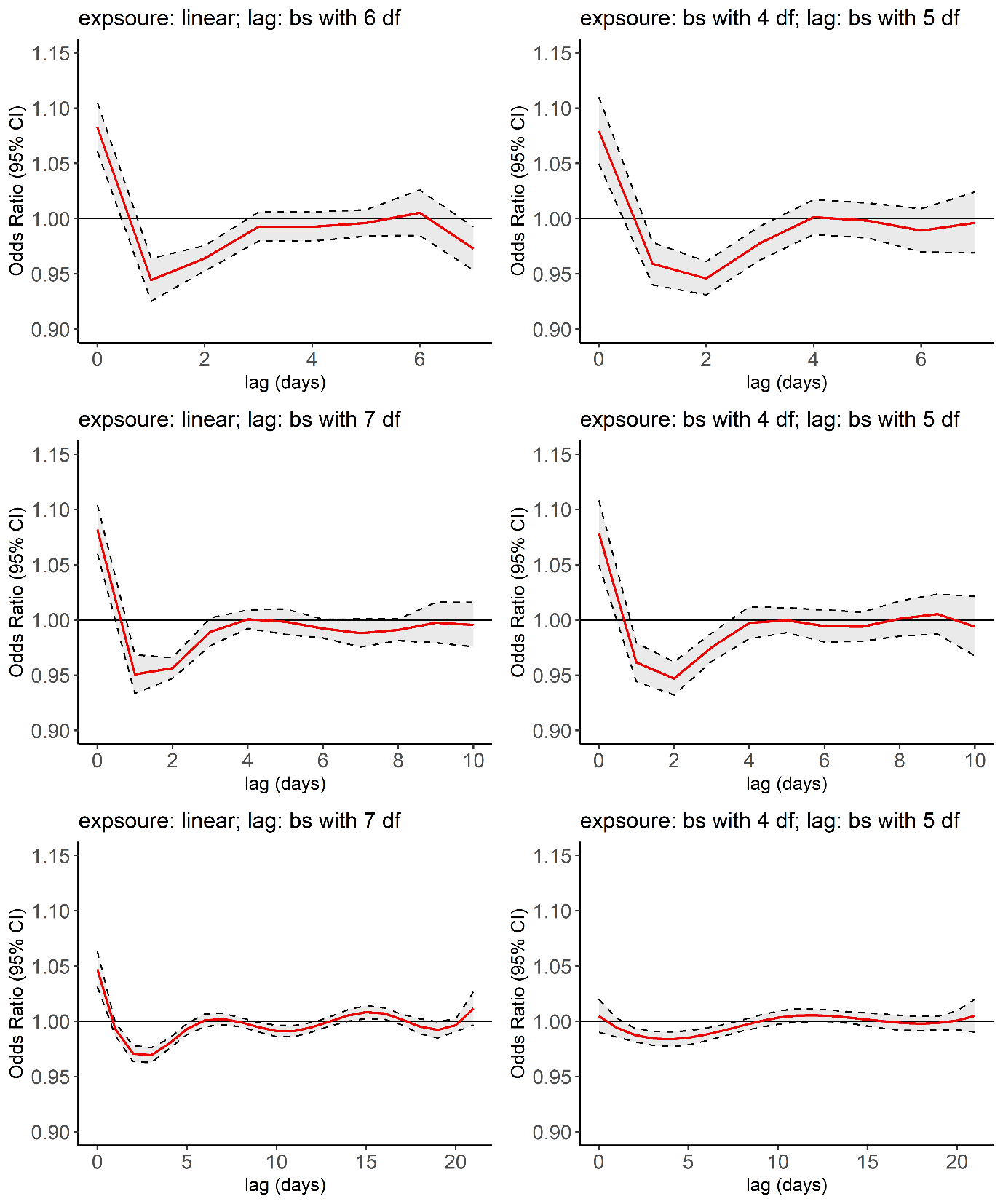


**Fig. S1**. Sensitivity analyses examining different lag periods (0–7, 0–10, and 0–21 lag days) and spline types, including a B-spline (bs) with 4 degree of freedom (df) for the exposure-response effect and a B-spline with 5 df for the lag-response effect. Shaded grey areas represent the 95% confidence intervals.


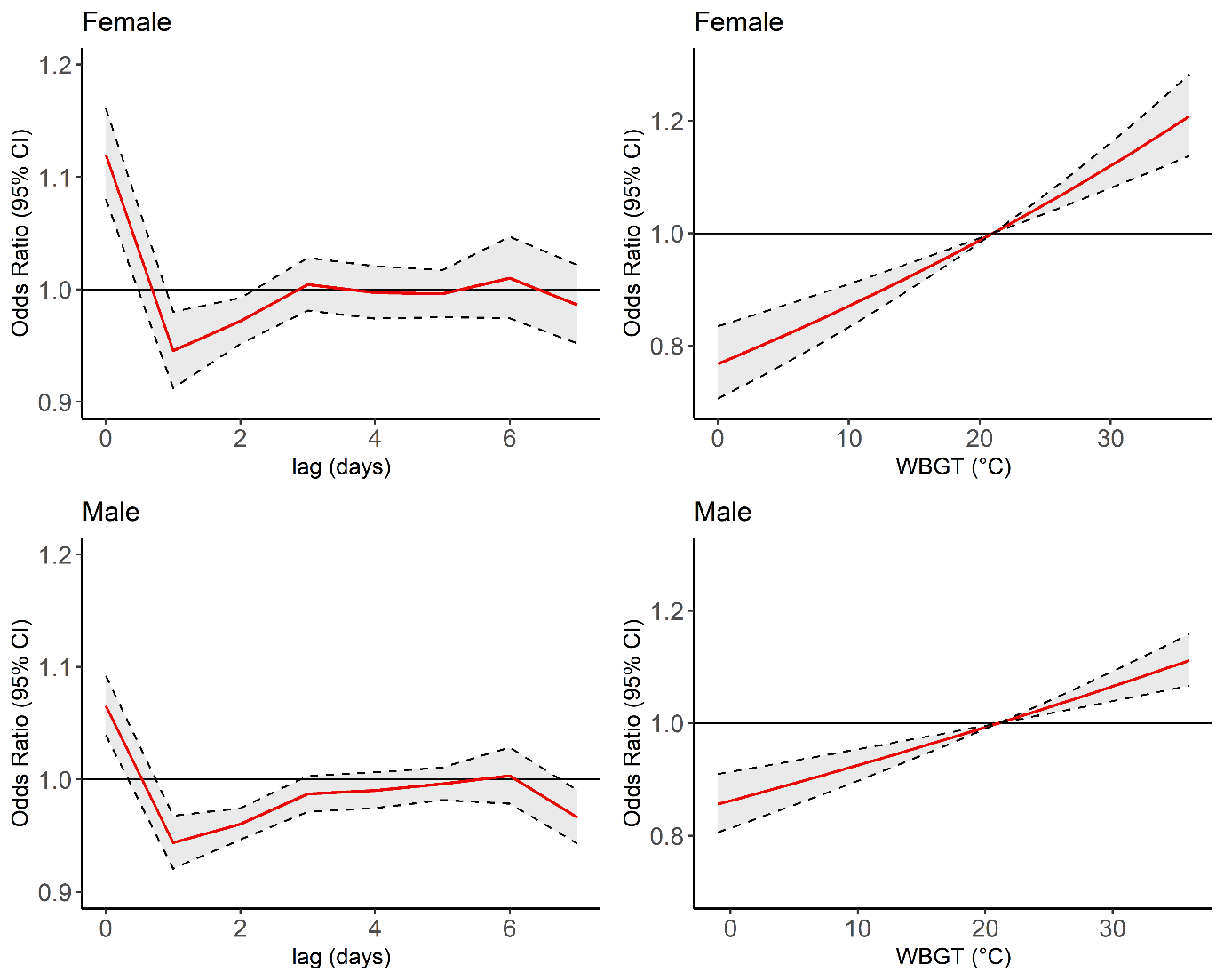


**Fig. S2.** Stratified analysis by sex. (Left panel) individual lag effects of wet-bulb globe temperature (WBGT) on epilepsy; (right panel) exposure-response relationships.


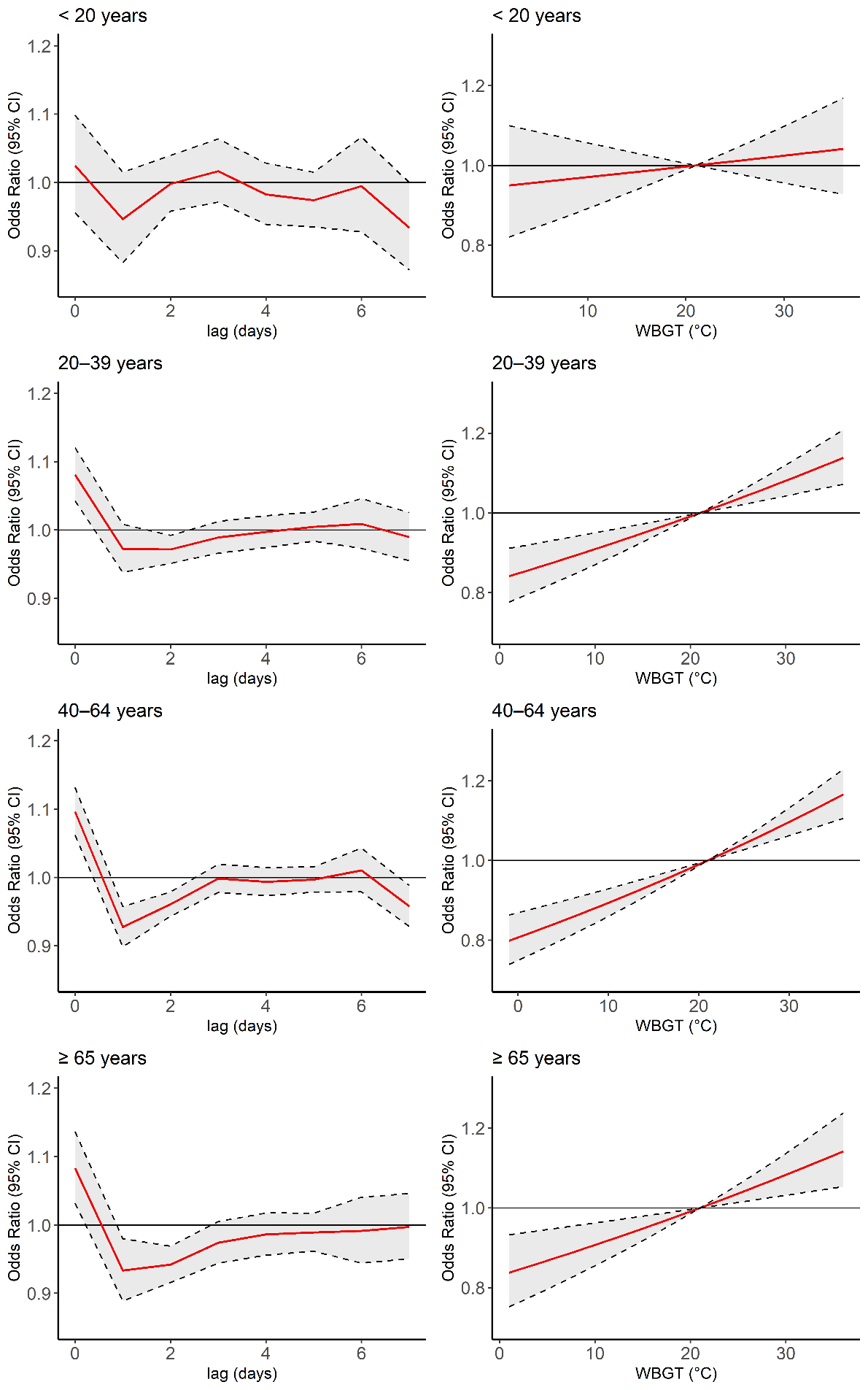


**Fig. S3.** Stratified analysis by age group. (Left panel) individual lag effects of wet-bulb globe temperature (WBGT) on epilepsy; (right panel) exposure-response relationships.


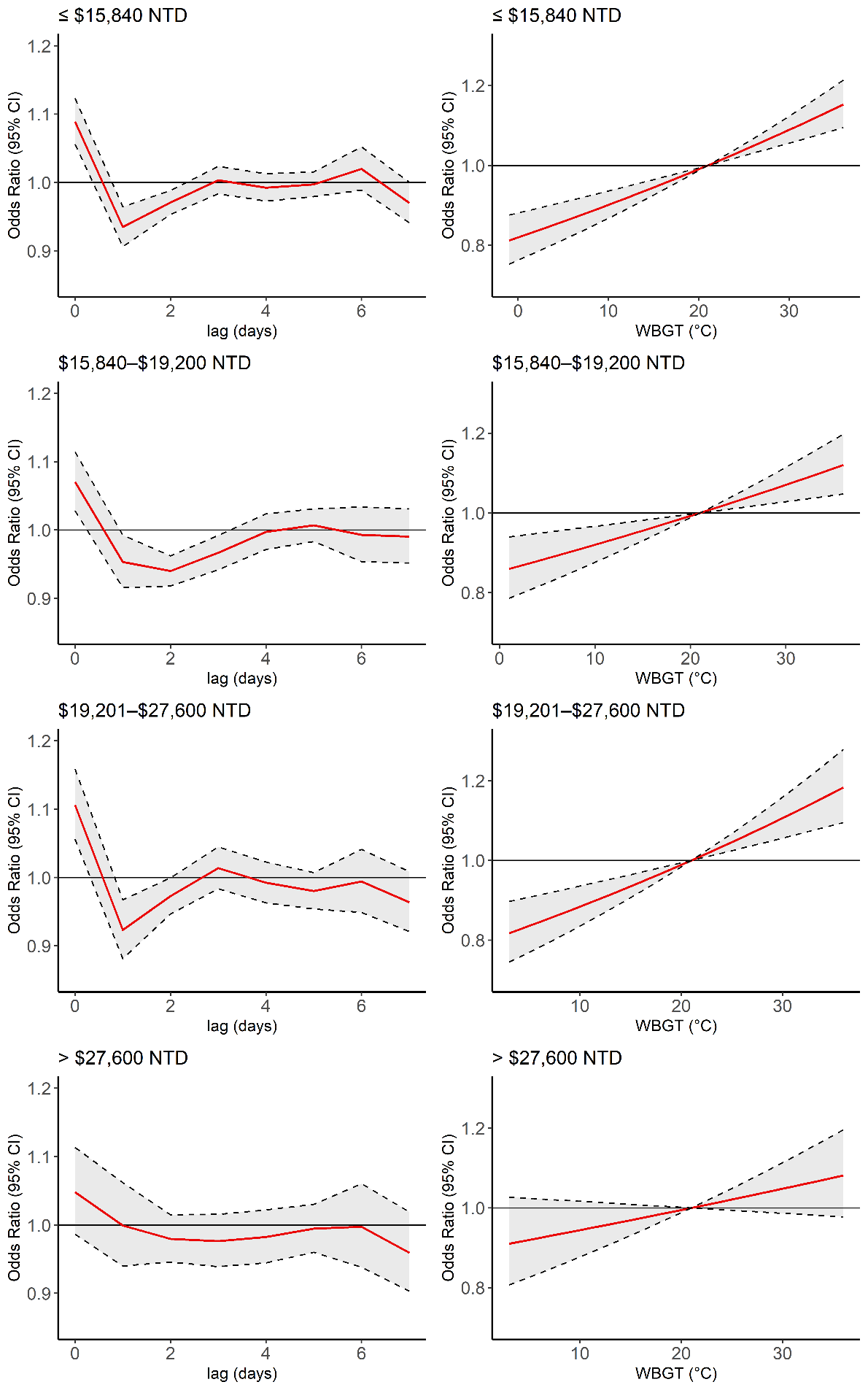


**Fig. S4.** Stratified analysis by socioeconomic status. (Left panel) individual lag effects of wet-bulb globe temperature (WBGT) on epilepsy; (right panel) exposure-response relationships.
